# Astrocytic EAAT‐2 Upregulation Supports Neuro‐Axonal Homeostasis in Chronic Inflammatory CNS Lesions

**DOI:** 10.1002/glia.70243

**Published:** 2026-10-23

**Authors:** Luise Reukauf, Anca Margineanu, Bakhrom Muinjonov, Jessy Chen, Yiqin Cui, Kamil Rosiewicz, Helena Radbruch, Ian Munro, Marlen Alisch, Volker Siffrin

**Affiliations:** ^1^ Experimental and Clinical Research Center (ECRC) Charité ‐ Universitätsmedizin Berlin and Max Delbrück Center for Molecular Medicine in the Helmholtz Association Berlin Germany; ^2^ Advanced Light Microscopy Max Delbrück Center for Molecular Medicine in the Helmholtz Association Berlin Germany; ^3^ Klinik für Neurologie und Experimentelle Neurologie Charité – Universitätsmedizin Berlin Berlin Germany; ^4^ Institute of Neuropathology Charité – Universitätsmedizin Berlin Berlin Germany; ^5^ Photonics Group, Physics Department Imperial College London London UK

**Keywords:** excitatory amino acid transporter (EAAT)–astrocyte, excitotoxicity, experimental autoimmune encephalomyelitis, multiple sclerosis, neuroprotection

## Abstract

Neuronal loss in multiple sclerosis (MS) is a key factor in the progression of disability. Axonal degeneration within MS lesions is central to progressive MS pathology, yet its mechanisms are not fully understood. Excess extracellular l‐glutamate (GLUT) is neurotoxic due to overstimulation of GLUT receptors. Excitotoxicity, driven by GLUT, is a suspected contributor to several central nervous system (CNS) diseases. Astrocytic glutamate transporters EAAT‐1 and EAAT‐2 play crucial roles in managing extracellular GLUT, yet their role in chronic MS remains underexplored. Using the animal model experimental autoimmune encephalomyelitis (EAE) and advanced microscopy in live anesthetized mice, we examined GLUT‐mediated neurotoxicity and protective mechanisms in chronic CNS lesions. By employing genetically encoded fluorescent biosensors, we visualized extracellular GLUT levels and axonal Ca^2+^ dynamics within the live mouse brain during EAE. In acute EAE, extracellular GLUT levels were high, while in chronic lesions, normalized GLUT levels together with sustained EAAT‐2 upregulation were consistent with a protective astrocytic response. EAAT‐2 inhibition in chronic lesions led to higher intra‐axonal Ca^2+^ levels and morphological changes consistent with neuroaxonal injury, indicating that EAAT‐2 upregulation may counter excitotoxicity and support neuroprotection. Post‐mortem MS brain tissue analysis provided complementary evidence, showing elevated EAAT‐1 and EAAT‐2 expression in chronic MS lesions with expression patterns varying according to lesion type and cellularity. This study highlights astrocytic mechanisms that may contribute to neuroaxonal protection in MS tissue and suggests targeting GLUT transporters as a promising strategy to limit neurodegeneration in progressive MS.

## Graphical abstract

Inflammatory CNS lesions increase extracellular glutamate. Astrocytic EAAT regulation controls glutamate clearance, stabilizes axonal Ca^2+^ homeostasis, and determines neurite survival during chronic neuroinflammation.

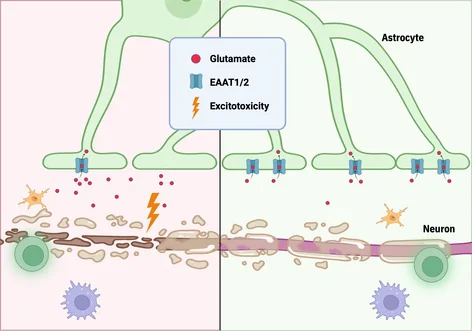

## 1 Introduction

Multiple sclerosis (MS) is the most common chronic inflammatory disease of the central nervous system (CNS) with more than 2.5 million people affected throughout the world. The underlying CNS pathology is that of invading peripheral immune cells associated with almost complete loss of myelin in focal lesions (Kuhlmann et al. 2017). The damage of the neuronal compartment is in relation to the demyelination much more subtle, but has been recognized as a major aspect of MS pathology (Trapp et al. 1998). Most importantly, the extent of loss of neurons and their processes is associated with accumulating disability in MS over the course of the disease (Singh et al. 2017; Elliott et al. 2019). The mechanisms of neurodegeneration in the context of inflammatory demyelination are still under controversial debate (Siffrin, Vogt, et al. 2010; Absinta et al. 2020). Reports detailing the neuronal damage processes and evidence from direct imaging in chronic MS lesions are rare. Longitudinal visualization of myelin damage can be routinely applied in MS patients using magnetic resonance imaging (MRI) (Brex et al. 2002; Fisniku et al. 2008), but neurodegeneration and the underlying pathophysiological processes are much more difficult to explore (Rocca et al. 2015). Imaging in the experimental autoimmune encephalomyelitis (EAE) animal model, which replicates certain aspects of MS, provides tools to link clinical symptoms with the molecular mechanisms driving inflammatory neurodegeneration (Siffrin, Radbruch, et al. 2010; Nikić et al. 2011; Witte et al. 2019). These investigations benefit from the capability to generate various transgenic animals expressing fluorescent biosensors and from advanced two‐photon microscopy techniques. Therefore, in this contribution we correlated the results of intravital microscopy with those obtained with fluorescent immunolabeling in EAE and human MS tissues to obtain further insights into the progressive neurodegeneration mechanism in the chronic phase of MS.

We focus on excitotoxicity—a neurodegeneration mechanism discussed throughout neurological disease entities, which is mediated by l‐glutamate (GLUT). In acute relapses, increased levels of GLUT have been reported in the cerebrospinal fluid of persons with MS (pwMS) (Sarchielli et al. 2003), while remission was associated with unobtrusive cerebro‐spinal fluid (CSF) GLUT concentrations (Stover et al. 1997). GLUT is the major activating neurotransmitter at synapses in the CNS with an essential role for neuronal network activity and neuronal survival. However, excessive extrasynaptic GLUT receptor activation leads to neurotoxicity or more specifically: excitotoxicity (Parsons and Raymond 2014). Control of extracellular GLUT levels in the CNS is the genuine task of astrocytes which express the two major subtypes of amino acid transporters: the excitatory amino acid transporter 1 (EAAT‐1, also called glutamate aspartate transporter 1 (GLAST)) which is encoded by the *SLC1A3* gene and the excitatory amino acid transporter 2 (EAAT‐2, also called glutamate transporter 1 (Glt‐1)) encoded by the *SLC1A2* gene. Though explored in many of the primary neurodegenerative CNS disorders in which these transporters are mostly decreased, for MS there is only scarce information available.

In our study, we aimed to expand the evidence accumulating so far on the excitotoxicity and the role of astrocytic EAATs in neuroinflammatory lesions by direct visualization in intravital experiments of EAE mice. To achieve this, we monitored the lesional foci in EAE within the live mouse brain, visualized the local glutamate levels as well as the neuro‐axonal Ca^2+^ response and the response to pharmacological drugs modulating the EAATs' activity. The aim was to investigate the role of astrocytic EAATs in protection against neurodegeneration during the chronic phase of the EAE model disease and explore the relevance of these findings in post‐mortem brain tissue derived from persons with progressive MS.

## 2 Results

### 2.1 Chronic EAE Lesions Show Reduced Immune Cell Infiltration, Increased Astrogliosis, and Similar Neurofilament Expression to Acute Lesions

First, we characterized chronic inflammatory lesions in experimental autoimmune encephalomyelitis (EAE) comparing this stage with acute EAE (3–7 days after onset of symptoms). Chronic EAE was defined as disease duration > 14 days after symptom onset; the chronic lesions analyzed histologically in this study were obtained ≥ 30 days after symptom onset. Acute EAE was associated with higher disability, as reflected by higher clinical scores, with peak severity occurring 2–3 days after symptom onset, followed by gradual recovery (Figure 1a). Animals typically reached peak disease severity 2–3 days after the onset of clinical signs and showed gradual improvement over the subsequent days to weeks; chronic EAE was defined as disease duration exceeding 14 days after symptom onset. We characterized the inflammatory areas in the spinal cord sections derived from acute and chronic EAE and compared them with normal appearing white matter (NAWM) control areas within the same section and with spinal cord sections derived from naïve control mice (no immunization). Immunofluorescence staining was performed on spinal cord cross‐sections (Figures 1b and S1). Analysis of CD45+ cells (leukocytes) was used to visualize the distribution of the peripherally derived immune cells/activated microglia in context with glial fibrillary acidic protein (GFAP) IF staining as marker for astrocytes (Figure 1b). In naïve spinal cord sections, hardly any CD45+ cells were identified. In acute EAE, dense infiltrates of CD45+ cells were identified in focal lesions within the white matter of the spinal cord. In chronic EAE, CD45+ immune cells are still present, but less numerable than in the acute inflammation state concurrent with increased GFAP fluorescence intensity indicating reactive astrocytes. Astrogliosis and a moderate immune cell infiltration into the CNS parenchyma were described in chronic lesions in MS (Kuhlmann et al. 2017). Additional MBP staining provided complementary information on myelin integrity within EAE lesions (Figure S1). As demyelination in EAE was incomplete and heterogeneous, lesion areas were primarily identified based on inflammatory infiltration, increased cellularity, and reactive gliosis rather than MBP loss alone.

**FIGURE 1:**
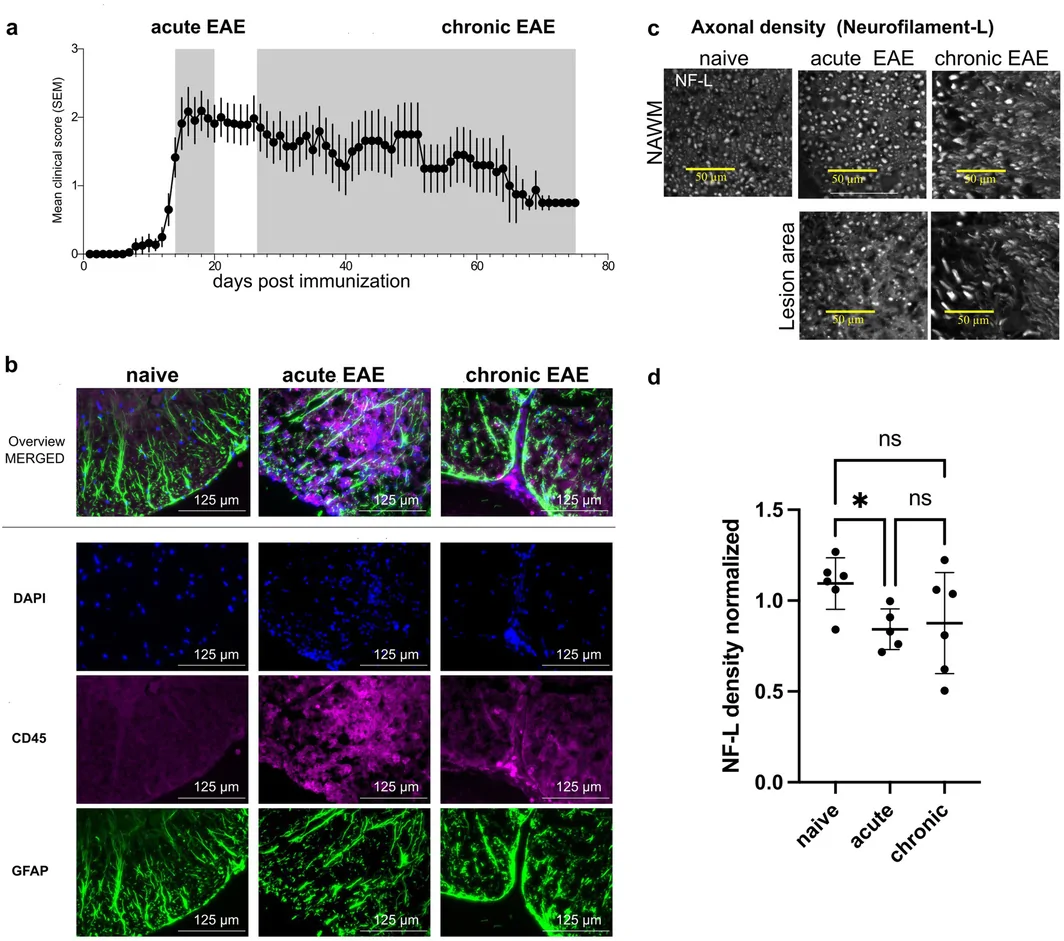
Comparison of lesion morphology and axonal loss in acute and chronic EAE spinal cords and in control mice. (a) Clinical score (mean ± SEM) of EAE mice (*n* = 20, pooled animals from at least three independent experiments). Shaded in gray is the analysis window that is, the time frame of acute versus chronic EAE stage, at which the mice were either examined with two‐photon microscopy or prepared for immunofluorescence staining. (b) Overview of exemplary spinal cord cross‐sections from a control naïve mouse, acute (Day 17) and chronic (Day 72) EAE mice showing the DAPI (blue), CD45 (magenta), and GFAP (green) distribution (40× magnification). The upper row shows merged images of control spinal cord (naive), acute, and chronic EAE of the three spectral channels, while Rows 2–4 show individual channel images of DAPI (2nd row), CD45+ (3rd row), and GFAP (4th row). The lesion areas are characterized by the migration of CD45+ immune cells into the white matter of the spinal cord. GFAP is upregulated in chronic EAE. (c) NF‐L IF of representative spinal cord cross‐sections from naïve mice, white matter acute and chronic EAE lesions, with respective normal appearing white matter (NAWM) areas within the same section of EAE lesions. (d) Quantification of NF‐L immunofluorescence (IF) in naïve, acute, and chronic EAE mice. For each animal, NF‐L IF density in the lesion area (LA) was normalized to that of the corresponding normal‐appearing white matter (NAWM) of the same section, yielding one data point per animal (naïve: *n* = 6; acute: *n* = 5; chronic: *n* = 6). In naïve mice, two anatomically comparable white matter regions were used for normalization. Statistical analysis was performed using Welch's one‐way ANOVA followed by Dunnett's T3 multiple comparisons test. Each dot represents one animal; bars indicate mean ± SD. Significance levels: *p < 0.05. Non‐significant comparisons are shown.

In these EAE lesions, we analyzed the presence and preservation of axons between control, acute and chronic EAE lesions. Spinal cord sections were stained against neurofilament light (NF‐L), a pan axonal marker. In EAE cross‐sectional spinal cord tissue samples, we analyzed focal areas of immune cell infiltration (“lesion area, LA”) by the abundance of DAPI, CD45 and GFAP IF in the white matter. These LA were compared with a normal‐appearing white matter (NAWM) control area (CA) within the same section, while in naïve control animals, two anatomically comparable white matter regions per section were analyzed (Figure 1c).

In both acute and chronic EAE lesion areas, NF‐L immunofluorescence was reduced relative to the corresponding intra‐animal reference regions, reaching statistical significance compared with naïve controls in acute EAE. NF‐L density in chronic EAE lesions was more heterogeneous and did not differ significantly from either naïve or acute EAE animals (Figure 1d). Thus, while the data demonstrate reduced axonal density during acute EAE, they do not provide evidence for a significant change in axonal density between the acute and chronic disease stages.

### 2.2 Chronic EAE Lesions Exhibit Reduced Levels of Extracellular GLUT

We evaluated the free GLUT levels in acute and chronic EAE by using intravital two‐photon laser scanning microscopy (TPLSM) of living anesthetized mice. We cross‐bred transgenic mice expressing the inducible glutamate sensor (iGluSnfr (Madisen et al. 2015) x R26‐LNL‐tTA (Wang et al. 2008) with a reporter strain for neurons Nex‐Cre (Goebbels et al. 2006); biosensors are also explained in Figure S2). GLUT binding to the extracellularly expressed transgenic construct on the neuronal membranes increases its fluorescence intensity (FI). Analysis of the FI in naïve mice identified very low levels of free GLUT in steady‐state conditions (Figure 2a). To monitor the peripheral immune cells in these animals in the context of GLUT dynamics, chimeric mice with bone marrow from red fluorescent mice were generated (Siffrin, Radbruch, et al. 2010). These tdRFP‐ > Nex‐Cre × iGluSnFR mice (red immune cells, GLUT sensor in axons/neurons) were immunized for active EAE and TPLSM was conducted in both acute and chronic EAE stages in anesthetized animals at the lower brainstem level as previously described for up to 6 h (Siffrin et al. 2009, 2015; Siffrin, Radbruch, et al. 2010). The microscope setup is shown in Figure S2. In acute EAE, increased fluorescence intensity (FI) indicated elevated GLUT levels in the context of pronounced immune cell infiltration (Figure 2a). At higher magnification, axonal segments within acute lesions exhibited focal increases in GLUT sensor fluorescence (Figure 2b). Absolute reporter signals were low (Δ*F*/*F* ~1.2–1.8), likely reflecting the limited dynamic range and/or sensitivity of the reporter system (Aggarwal et al. 2023). When quantified across image stacks, mean fluorescence intensity (MFI) was higher in acute EAE lesions compared with control tissue, whereas in chronic EAE lesions only focal signal increases were detectable, mainly in proximity to immune cell infiltrates. Accordingly, overall MFI values in chronic lesions were comparable to those observed in control animals (Figure 2c). Finally, MFI values showed a positive correlation with clinical EAE scores (Figure 2d), indicating an association between GLUT sensor signal intensity and disease severity. Given the limited number of animals in the acute EAE imaging cohort (*n* = 2), comparisons between acute and chronic disease stages are considered descriptive and were not subjected to inferential group statistics.

**FIGURE 2:**
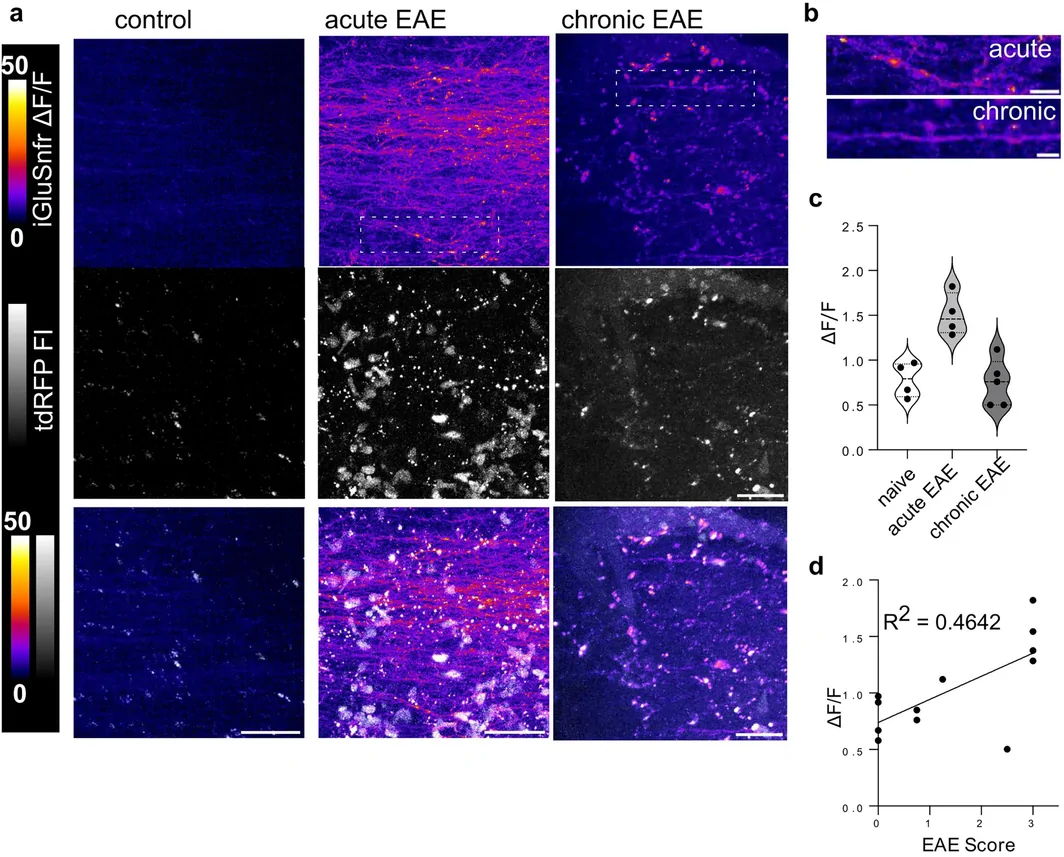
In vivo GLUT levels in naïve brainstem, acute and chronic EAE lesions. Intravital two‐photon laser scanning microscopy (TPLSM) was conducted on tdRFP‐ > Nex‐Cre × iGluSnFR mice, which have red fluorescent immune cells and the GLUT sensor expressed in axons and neurons. (a) The top row shows GLUT sensor fluorescence intensity images (“Fire” look‐up table color‐coded, 520/40 nm) in a naïve control mouse, acute and chronic EAE. The intensity images show the normalized values Δ*F*/*F*, where Δ*F* = pixel intensity—mean background intensity and *F* = mean background intensity. The middle row presents immune cell infiltrates (CD45+ cells in gray) within the respective imaging area, and the bottom row displays compound images combining immune cells with neuro‐axonal GLUT sensor visualization. Scale bar: 50 μm. (b) Higher‐magnification views of representative axonal segments from the fluorescence intensity images in (a), showing acute (upper) and chronic (lower) EAE lesions. Scale bar: 20 μm. (c) Quantification of the mean fluorescence intensity (MFI) of image stacks (consisting of 8–10 *z* planes) was performed across *n* = 3 control mice, *n* = 2 acute EAE mice, and *n* = 5 chronic EAE mice. Each data point represents an imaging area and a mean of 10 time points. (d) Correlation between the mean ΔF/F of individual image stacks and the respective clinical score of the EAE mice is depicted.

### 2.3 Intravital Imaging Reveals Focal Axonal Ca^2+^ Elevations Associated With Neuro‐Axonal Damage in Chronic EAE

Because the GLUT reporter mice suggested a potential contribution of glutamate‐related toxicity to axonal damage but lacked sufficient sensitivity for mechanistic analyses, we next employed our previously established neuro‐axonal Ca^2+^ indicator mice (Thy1‐CerTn‐L15 (Siffrin et al. 2009, 2015)). Using these mice, we investigated Ca^2+^ dynamics in neuro‐axonal structures within chronic EAE lesions, in the context of bone marrow chimerism generated from B6.tdRFP mice (Luche et al. 2007), which fluorescently label peripheral immune cells (Figure 3a). The genetically encoded biosensor CerTn‐L15 is based on a troponin‐derived Ca^2+^‐binding domain that undergoes a conformational change upon binding cytosolic free Ca^2+^ ([cCa^2+^]), resulting in altered Förster resonance energy transfer (FRET) between the fluorescent proteins Cerulean and YFP (Figure S1).

**FIGURE 3:**
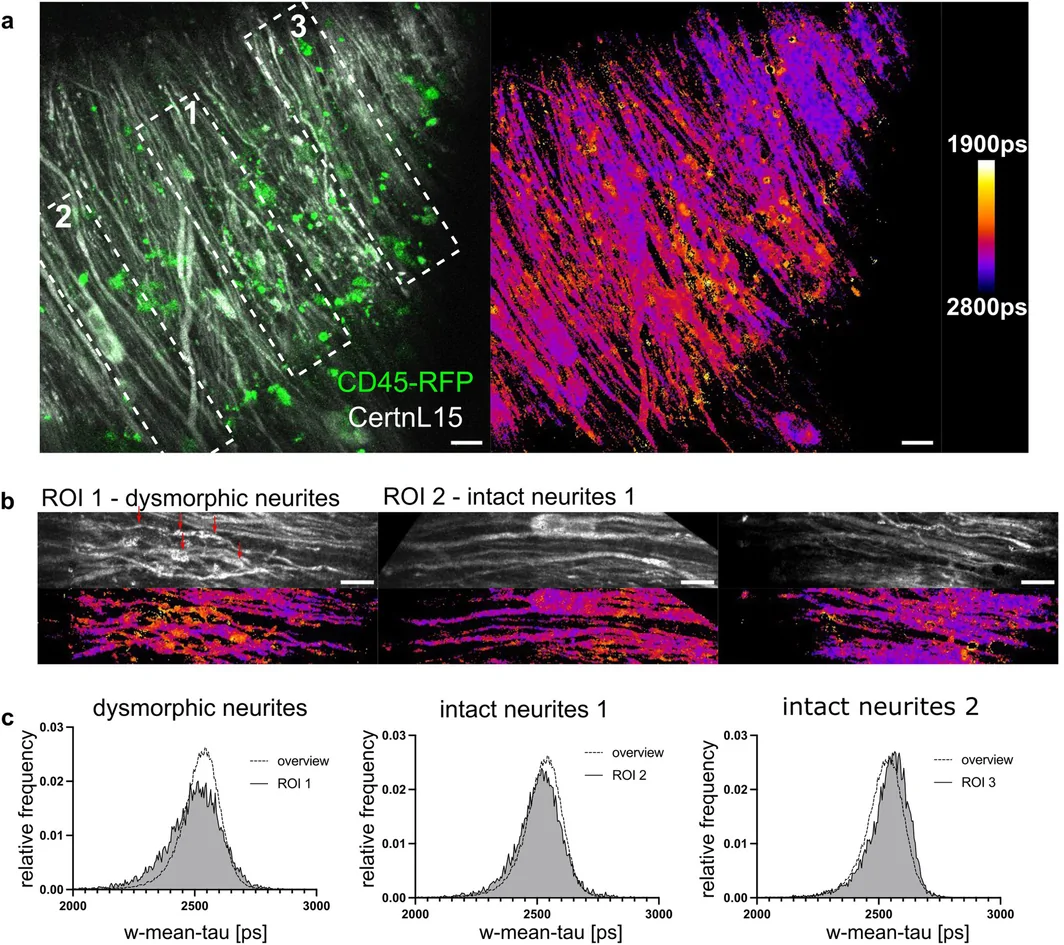
Intravital analysis of neuro‐axonal Ca^2+^ dynamics in chronic EAE lesions using Thy1‐CerTn‐L15 mice. (a) Representative intravital images of chronic EAE brainstem lesions in Thy1‐CerTn‐L15 mice with bone marrow chimerism from B6.tdRFP donors (Day 19 after symptom onset; score 0.75/maximal previous score: 2.5). Cerulean (CFP) intensity imaging delineates the morphology of neuro‐axonal structures (left panel, grayscale), whereas tdRFP fluorescence (green) labels infiltrating peripheral immune cells. Lesion areas were identified in vivo by the local accumulation of tdRFP‐labeled peripheral immune cells, indicative of inflammatory infiltration. The corresponding fluorescence lifetime imaging (FLIM) of Cerulean (right panel) visualizes neuro‐axonal Ca^2+^ dynamics independently of sensor expression level and tissue scattering, displayed as false‐color maps using an inverted “fire” color scale. (b) Representative fluorescence lifetime imaging (FLIM) maps of Cerulean in neurites within chronic EAE brainstem lesions, demonstrating localized reductions in fluorescence lifetime. Regions of interest (ROIs) selected for quantitative analysis are indicated in (a). Red arrows highlight morphologically abnormal (dysmorphic) neurites. (c) Quantitative analysis of Cerulean fluorescence lifetime distributions in morphologically intact and dysmorphic neurites (as defined by ROIs 1–3). Fluorescence lifetime is expressed as the intensity‐weighted mean lifetime (w‐mean‐tau, ps) derived from pixel‐wise multi‐exponential decay fitting and averaged over each ROI. ROIs containing dysmorphic axons show a shift toward shorter w‐mean‐tau values, reflecting reduced average fluorescence lifetimes within these neurites.

Cerulean intensity images were used to delineate the morphology of the neuro‐axonal compartment in context of RFP intensity imaging for the invading immune cells (Figure 3a left panel), whereas Ca^2+^ dynamics were assessed independently of expression level and tissue scattering by fluorescence lifetime imaging (FLIM) of Cerulean (Figure 3a right panel), as previously described (Figure S2; Rinnenthal et al. 2013; Siffrin et al. 2015). A reduction in the fluorescence lifetime of Cerulean (detected at 475/50 nm) reflects increased cytosolic Ca^2+^ levels and was observed in neurites within chronic EAE brainstem lesions (Figure 3b), with dysmorphic axons showing a pronounced leftward shift toward shorter lifetimes (Figure 3c). This approach enables intravital, high‐resolution interrogation of focal neuro‐axonal Ca^2+^ dysregulation as a mechanistic component of axonal damage formation in chronic EAE (Figure S3a,b).

### 2.4 EAAT Blockade and Intra‐Axonal Ca^2+^ Dynamics in Chronic EAE

To functionally probe the contribution of glutamate uptake to neuro‐axonal Ca^2+^ homeostasis in chronic EAE lesions, we performed two‐photon intravital time‐lapse imaging in Thy1‐CerTn‐L15 mice during the chronic disease stage (Figure 4a). After a stable baseline recording period of at least 20 min, selective pharmacological inhibitors of EAAT‐1 and EAAT‐2 were applied. EAAT‐1 was inhibited using UCPH‐101 (Abrahamsen et al. 2013), a selective non‐substrate inhibitor (Figure 4b), whereas EAAT‐2 function was blocked by topical application of dihydrokainate (DHK) (Jensen and Bräuner‐Osborne 2004), a kainate derivative with relative selectivity for EAAT‐2 (Figure 4c).

**FIGURE 4:**
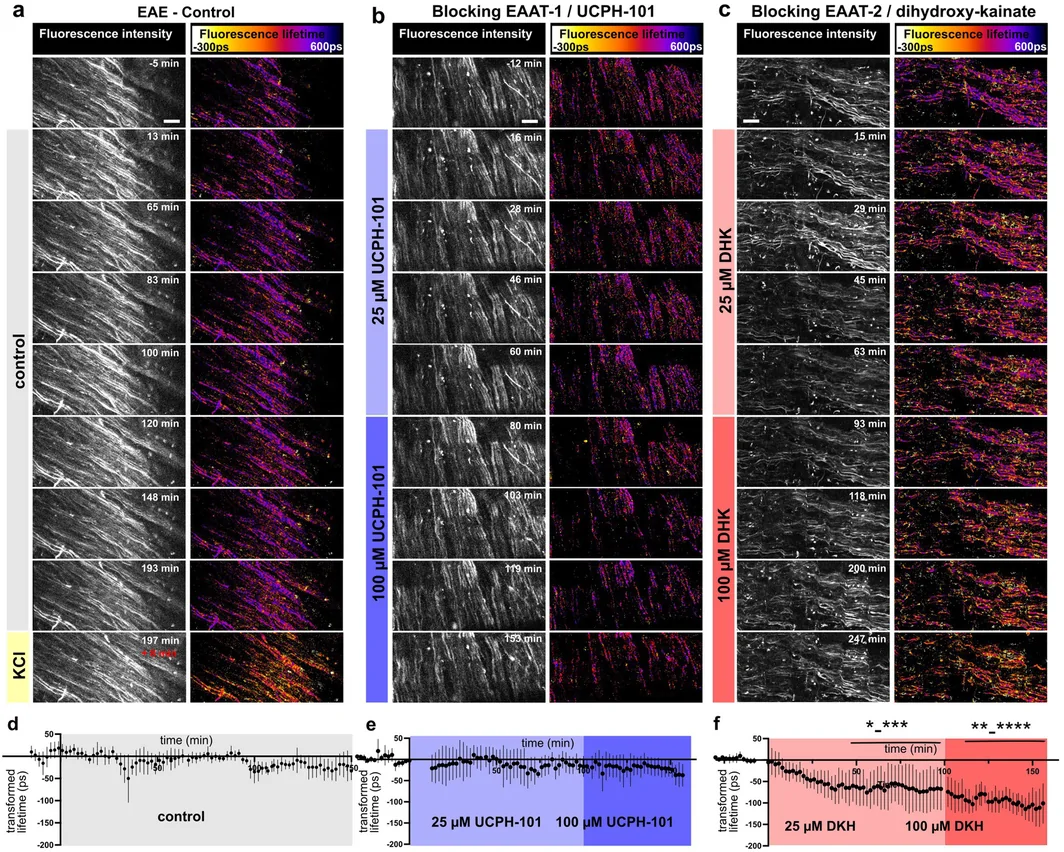
Effect of EAAT‐1 and EAAT‐2 blocking agents on the intracellular Ca^2+^ signaling within the mouse brain stem monitored via two‐photon intravital microscopy. (a) Time‐lapse images in a chronic EAE phase mouse showing the fluorescence intensity of Cerulean (FI, gray, left column) and FLIM images (false colors, “Fire” look‐up table, right column) of the FRET Ca^2+^ biosensor expressed in the axons of the mouse. FLIM data show intra‐axonal [Ca^2+^] dynamics as changes of the biosensor's lifetime. For comparability of different records, raw lifetimes within one recording were normalized to the mean of the median lifetimes measured during a 20 min baseline before the drug application. KCl (1%) was added at the end of the imaging sequence of the control mouse to confirm that the FRET biosensor's lifetime is indeed reduced when the intracellular [Ca^2+^] increases (last image, bottom). Scale bar: 20 μm. (b) Time‐lapse images (similar analysis as in A) in a chronic EAE mouse with application of different concentrations of the EAAT‐1 blocking agent UCPH‐101, which was topically administered during imaging after 20 min of baseline recording. Intra‐axonal [Ca^2+^] dynamics (right column) are shown as lifetime values of the biosensor in relation to the anatomical structures (FI of the biosensor, left column). Scale bar: 20 μm. (c) Time‐lapse images (similar analysis as in A) in a chronic EAE mouse with application of different concentrations of the EAAT‐2 blocking agent dihydroxy‐kainate (DHK), which was topically administered during imaging after 20 min of baseline recording. Intra‐axonal [Ca^2+^] dynamics (right column) are shown as lifetime changes of the biosensor in relation to the anatomical structures (FI of the biosensor, left column). Scale bar: 20 μm. (d‐f) Graphs illustrating the time variation of the Cerulean lifetime of the FRET [Ca^2+^] biosensor over the duration of the experiments in control EAE mice and mice treated with EAAT‐1 and EAAT‐2 blocking agents. A baseline measurement was taken for a specified duration before drug application, and all lifetime values were transformed by subtracting the mean of the baseline median lifetime. Means and standard deviations were calculated using 2–3 imaging areas for *n* = 6 EAE control mice (d), *n* = 3 mice treated with UCPH‐101 (e), and *n* = 3 mice treated with DHK (f). Reduction in the mean lifetime values indicates an increase in the intra‐axonal [Ca^2+^]. Statistical analysis: Multiple Mann–Whitney tests were performed comparing treatments with control. *p < 0.05; **p < 0.005; ***p < 0.001; ****p < 0.0001.

In control EAE mice without pharmacological intervention, Cerulean fluorescence lifetimes remained stable throughout the imaging period, indicating unchanged intra‐axonal Ca^2+^ levels under baseline chronic EAE conditions (Figure 4a,d). Similarly, inhibition of EAAT‐1 by UCPH‐101 did not induce significant changes in Cerulean lifetime trajectories, suggesting that EAAT‐1 blockade does not measurably affect intra‐axonal Ca^2+^ dynamics in chronic EAE lesions (Figure 4b,e). In contrast, blockade of EAAT‐2 resulted in a pronounced reduction of Cerulean fluorescence lifetime, reflecting an increase in intra‐axonal cytosolic Ca^2+^ (Figure 4c,f). This Ca^2+^ elevation developed after DHK application and was accompanied by morphological alterations of neurites, including features consistent with axonal damage. Quantitative analysis across imaging regions confirmed a significant decrease in mean lifetime values following EAAT‐2 inhibition compared with control conditions (Figure 4f).

Importantly, topical application of EAAT‐1 or EAAT‐2 blockers in naïve mice did not induce detectable changes in intra‐axonal Ca^2+^ levels, indicating that the observed effects are specific to the chronic inflammatory lesion environment (Figure S3c). Together, these findings indicate that sustained EAAT‐2 activity in chronic EAE lesions contributes to the stabilization of intra‐axonal Ca^2+^ homeostasis and may protect axons from ongoing glutamate‐related dysregulation under chronic inflammatory conditions.

### 2.5 Chronic EAE Lesions Are Characterized by Sustained Upregulation of the Glutamate Transporter EAAT‐2

We next examined the expression of the astrocytic glutamate transporters EAAT‐1 and EAAT‐2, the primary mediators of extracellular glutamate clearance in the CNS [5], in spinal cord tissue from naïve mice and from mice with acute and chronic EAE. In EAE spinal cord sections, 9–12 focal lesion areas within the white matter identified based on DAPI, CD45, and GFAP immunoreactivity, and corresponding contralateral normal‐appearing white matter regions within the same section were used as internal controls (Figure 5a). In naïve mice, two anatomically opposing white matter regions per section were analyzed. Quantitative analysis revealed that EAAT‐1 immunofluorescence intensity was significantly increased in acute EAE lesion areas compared with contralateral control regions, whereas no difference was detected in chronic EAE lesions (Figure 5b). In contrast, EAAT‐2 showed a robust and sustained increase in immunoreactivity in both acute and chronic EAE lesions relative to control regions (Figure 5c). Thus, while both glutamate transporters are upregulated during acute lesion formation, only EAAT‐2 remains persistently elevated in chronic EAE lesions.

**FIGURE 5:**
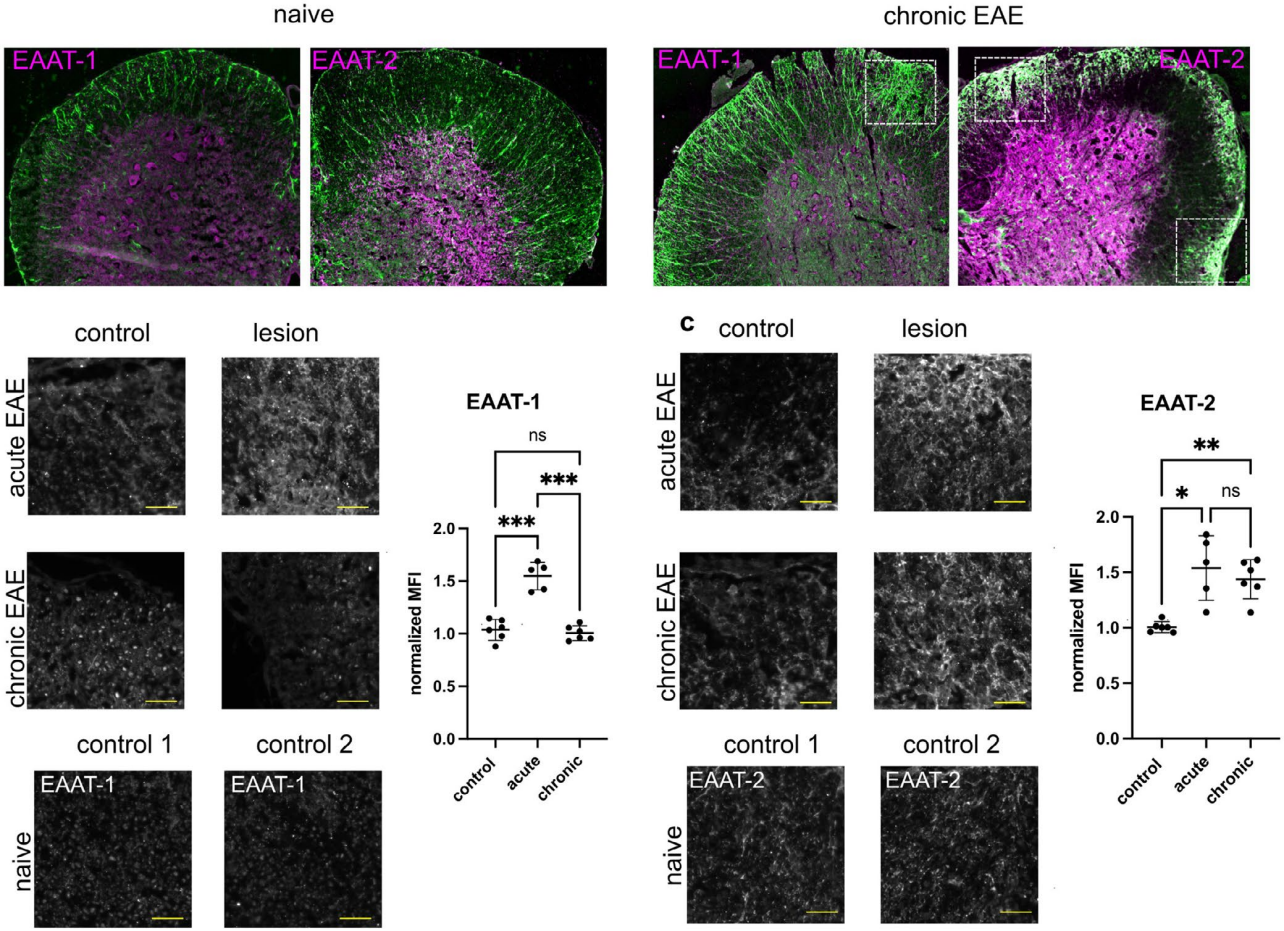
Quantification of EAAT‐1 and EAAT‐2 expression in acute and chronic EAE lesions. Quantification of EAAT‐1 and EAAT‐2 expression in acute and chronic EAE lesions. (a) Representative immunofluorescence overviews of EAAT‐1 or EAAT‐2 (magenta) and GFAP (green) in spinal cord sections from naïve and chronic EAE mice. The dotted box indicates a focal lesion area. Regions of interest (ROIs; 158 × 158 μm) were defined within lesion areas (LA) and corresponding contralateral normal‐appearing white matter control areas (CA) in the same section. (b) Left: Immunofluorescence staining of EAAT‐1 (grayscale) in representative ROIs from naïve, acute EAE (17 days post‐immunization), and chronic EAE (72 days post‐immunization) spinal cord sections acquired at 40× magnification. Lesion areas are shown adjacent to their respective control areas. Right: Fluorescence intensities were normalized to the median of the corresponding contralateral control area (CA); graphs show the mean of regional medians per mouse. Each dot represents one mouse. (c) Left: Immunofluorescence staining of EAAT‐2 (grayscale) in representative ROIs under identical conditions as in (b). Right: Corresponding quantification of EAAT‐2 fluorescence intensities as described in (b). Staining was performed on spinal cord sections from *n* = 6 naïve mice, *n* = 5 acute EAE mice, and *n* = 6 chronic EAE mice. Statistical analysis: Brown‐Forsythe and Welch ANOVA with Dunnett's T3 multiple comparisons test. *p < 0.05; **p < 0.01; ***p < 0.001; ns, not significant. Scale bar: 10 μm.

### 2.6 EAAT‐1 and EAAT‐2 Expression in Chronic MS Lesions Is Heterogeneous and Correlates With Lesion Cellularity

To assess the relevance of our experimental findings for human disease, we performed immunofluorescence analyses on post‐mortem brain tissue obtained from six individuals with longstanding progressive multiple sclerosis (pwMS; clinical and neuropathological characteristics in Table 1) harboring demyelinating white matter lesions.

**TABLE 1:** Clinical characteristics and neuropathological classification of multiple sclerosis (MS) cases included in the study.

Sample	MS type	Disease duration	Sex	Age	Years to EDSS 6	Years EDSS6 to death	Classification of the lesion by the NBB	Own classification
MS1	PP	16	m	44	7	9	Active demyelinating (Activity 3), inactive	Chronic inactive
MS2	SP	34	f	56	2	32	Reactive, active demyelinating (Activity 2), inactive	Chronic inactive
MS3	PP	31	f	54	8	23	Active demyelinating (Activity 2), chronic active demyelinating (Activity 3)	Mixed active/inactive
MS4	PP	14	m	41	2	12	Active demyelinating (Activity 3) inactive, gray matter, remyelinating	Chronic inactive
MS5	SP	25	m	49	5	20	Chronic active demyelinating (Activity 2), remyelinating	Mixed active/inactive
MS6	SP	22	m	44	11	11	Reactive, active demyelinating (Activity 2), inactive	Mixed active/inactive

*Note:* Shown are MS subtype (PP, primary progressive; SP, secondary progressive), disease duration, sex, age at death, time to EDSS 6, and time from EDSS 6 to death. Lesions were classified by the Netherlands Brain Bank (NBB) and additionally categorized using the authors' own lesion activity classification.

All lesions analyzed were identified by increased GFAP immunoreactivity (Figure 6a,b) together with reduced myelin basic protein (MBP) staining on adjacent sections (Figure S4), consistent with chronic demyelinated lesions.

**FIGURE 6:**
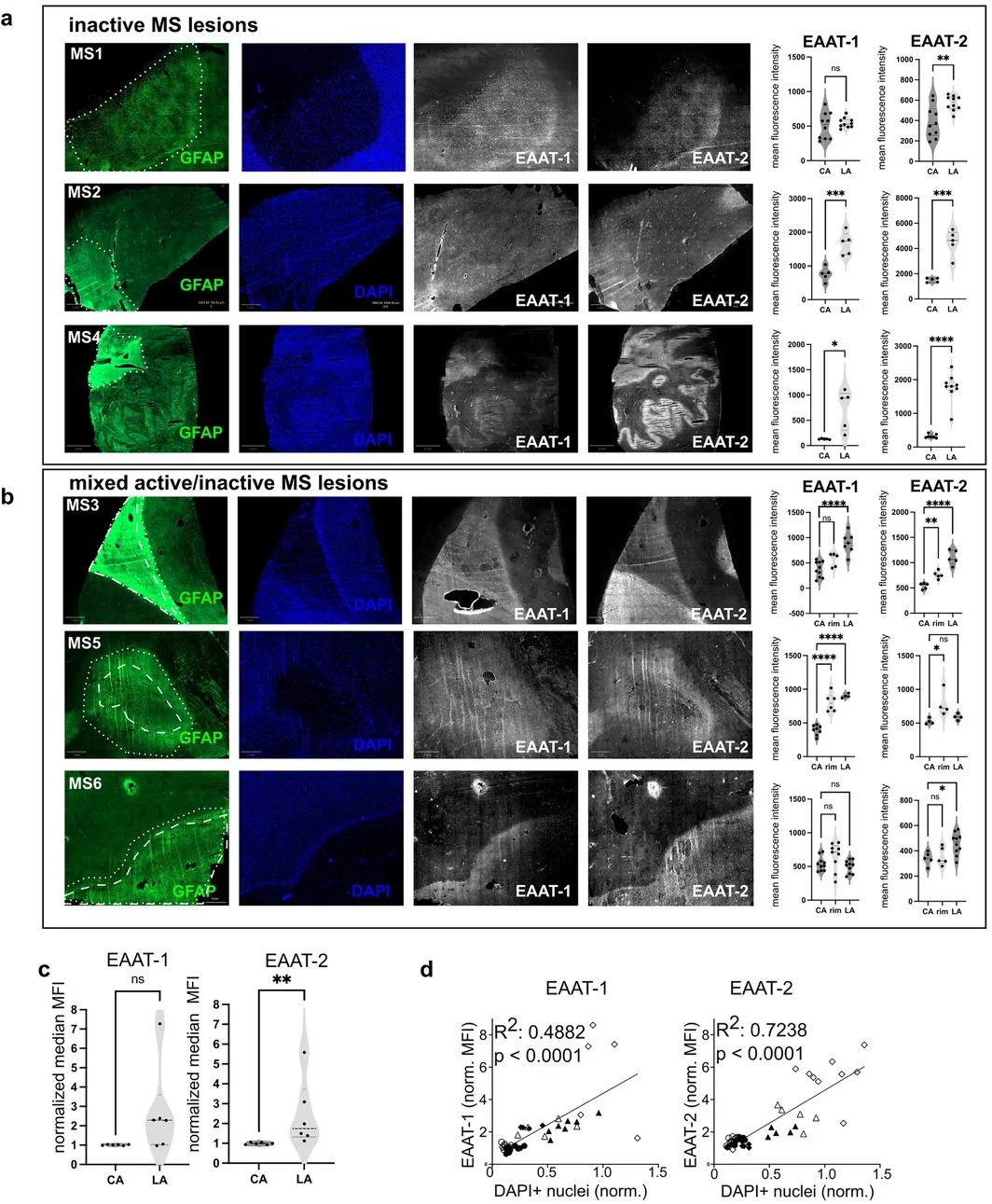
Heterogeneous EAAT‐1 and EAAT‐2 expression across chronic MS white matter lesions. Immunofluorescence analysis of EAAT‐1 and EAAT‐2 in chronic white matter lesions from individuals with progressive multiple sclerosis (pwMS). Tissue sections were stained for GFAP, DAPI, and either EAAT‐1 or EAAT‐2. Lesions were identified by increased GFAP immunoreactivity and reduced myelin basic protein (MBP) staining (Figure S4). Lesion activity was assessed by DAPI‐defined cellularity and the distribution of HLA‐DR‐positive microglia/macrophages (Figure S4), and lesions were classified as chronic inactive or mixed chronic active/inactive according to Kuhlmann et al. (2017). (a) Representative chronic inactive demyelinated lesions (*n* = 3) with dotted ROIs indicating lesion areas analyzed for EAAT‐1 and EAAT‐2 expression, compared with normal‐appearing white matter (NAWM). (b) Representative mixed chronic active/inactive lesions (*n* = 3), with analyses of lesion core (dotted ROI), cell‐rich lesion rim (dashed ROI), and NAWM. (c) Pooled analysis of NAWM‐normalized mean fluorescence intensity (MFI) of EAAT‐1 and EAAT‐2 across all MS cases. Individual dots represent patient‐level values calculated as the median MFI of every lesion ROIs (LA, CA) normalized to the corresponding NAWM ROIs (one lesion per patient). Statistical test: Mann–Whitney test, **p = 0.002. (d) Correlation of EAAT‐1 and EAAT‐2 expression with cellular density, assessed by DAPI‐positive nuclei per ROI. Scale bar: 1 mm. Statistical analysis: Mann–Whitney test (two groups) and Kruskal–Wallis test (three groups). LA, lesion area; NAWM, normal‐appearing white matter.

Lesion activity was further assessed by cellularity (DAPI staining) (Figure 6) and the distribution of HLA‐DR‐positive microglia/macrophages (Figure S4), allowing classification into inactive and mixed active/inactive lesions according to established criteria (Kuhlmann et al. 2017). Chronic inactive lesions are characterized by low cellularity and absence of a cell‐rich HLA‐DR‐positive lesion rim, and mixed chronic active/inactive lesions are characterized by a cell‐dense lesion edge enriched in HLA‐DR‐positive microglia/macrophages.

Sections were stained for either EAAT‐1 or EAAT‐2 together with GFAP. Regions of interest (ROIs) were defined within lesional areas and compared to normal‐appearing white matter within the same tissue section (Figure S5a) and for each tissue sample (graphs in Figure 6a,b). EAAT‐1 and EAAT‐2 immunoreactivity predominantly localized to GFAP‐positive astrocytic structures, identifying astrocytes as the principal cellular compartment of transporter expression in the analyzed MS tissue (Figure S5b).

Quantitative analysis revealed that both EAAT‐1 and EAAT‐2 immunofluorescence intensities were increased in MS lesion areas compared with corresponding control white matter (Figure 6c). In contrast, no comparable increase in EAAT‐1 or EAAT‐2 expression was detected in white matter tissue derived from individuals with amyotrophic lateral sclerosis (ALS; Figure S4c), which served as a non‐MS neurological disease control.

Within MS lesions, EAAT‐1 and EAAT‐2 expression were heterogeneous and displayed a patchy distribution. Chronic inactive lesion areas, characterized by low cellularity and reduced numbers of DAPI‐positive nuclei, generally showed lower EAAT‐1 and EAAT‐2 immunoreactivity. In contrast, areas corresponding to mixed chronic active/inactive lesions, which retained higher cellular density, exhibited increased transporter expression (Figure 6a,b,d).

Across all analyzed MS lesions, EAAT‐1 and EAAT‐2 fluorescence intensities positively correlated with the number of DAPI‐positive nuclei per ROI (Figure 6d), indicating an association between astrocytic glutamate transporter expression and local lesion cellularity, which may, at least in part, reflect regional differences in astrocyte abundance.

Together, these findings show that EAAT‐1 and EAAT‐2 expression is heterogeneous across chronic MS white matter lesions, with higher transporter expression associated with increased lesion cellularity. The relatively modest differences between lesion areas and corresponding NAWM further suggest that MS NAWM may not represent an entirely unaffected reference compartment.

## 3 Discussion

Identifying the mechanisms behind axonal dysfunction, neurite damage, and factors promoting tissue preservation in chronic inflammatory lesions is crucial for addressing the accumulating disability in progressive MS. Our findings are consistent with a neuroprotective contribution of astrocytic EAATs, suggesting that reactive astrocytes may limit glutamate‐mediated neuro‐axonal stress through EAAT upregulation. Importantly, pharmacological inhibition of EAAT‐2 in chronic EAE lesions increased intra‐axonal Ca^2+^, supporting a functional role of EAAT‐2 in maintaining neuro‐axonal Ca^2+^ homeostasis under inflammatory conditions.

Using intravital imaging, we previously demonstrated in the animal model EAE that the axonal injury is reversible at the early stage of increased intraaxonal [Ca^2+^] by blocking NMDA receptors in acute EAE lesions (Siffrin, Radbruch, et al. 2010). Furthermore, blocking NMDA or AMPA/kainate receptors have been shown to improve EAE signs (Pitt et al. 2000; Basso et al. 2008). The new intravital imaging experiments presented here directly demonstrate that the extracellular GLUT levels rise in acute lesions of EAE animals. These elevated levels correlate with high disease scores, whereas chronic lesions show lower extracellular glutamate levels together with sustained EAAT‐2 upregulation and lower disease scores.

Elevated extracellular glutamate is a well‐established driver of excitotoxicity, with astrocytic EAAT‐1 and EAAT‐2 serving as the primary machinery maintaining extracellular glutamate below excitotoxic levels in the CNS (Rothstein et al. 1996; Anderson and Swanson 2000). Under physiological conditions, the CNS white matter is comparatively low in EAATs relative to cortical areas, most likely reflecting the lower levels of glutamate release in this compartment. In the spinal cord, EAAT‐1 is expressed by only a small number of patchily distributed astrocytes (Winchenbach et al. 2016; Rosiewicz et al. 2020), while EAAT‐2 shows a broader, more pan‐astrocytic distribution but similarly remains at low levels in the white matter, with highest expression concentrated in the gray matter (Lepore et al. 2011; Yang et al. 2011). The upregulation of both transporters we observe in acute EAE white matter lesions therefore represents a marked induction against a low baseline—and the sustained EAAT‐2 elevation specifically in chronic lesions (Figure 5) suggests a lesion‐specific astrocytic adaptation that persists beyond the acute inflammatory phase. The predominant role of EAAT‐2 over EAAT‐1 in this context is consistent with data from genetic knockout models: EAAT‐2‐deficient mice developed gray matter pathology early in life, with increased epileptic seizure activity associated with uncompensated increased glutamate levels at synapses. These mice died prematurely and showed signs of neurodegeneration (Tanaka et al. 1997), while EAAT‐1‐deficient mice show only subtle deficits in complex coordination tasks, which fits to its pronounced cerebellar expression pattern (Watase et al. 1998). This dissociation is directly mirrored by our pharmacological experiments, in which EAAT‐2 blockade with DHK, but not EAAT‐1 blockade with UCPH‐101, increased intra‐axonal Ca^2+^ in chronic EAE lesions (Figure 4), suggesting that EAAT‐1 cannot compensate for EAAT‐2 loss in this inflammatory setting and that EAAT‐2 plays a specific and indispensable role in glutamate clearance within chronic lesions. However, as DHK inhibits EAAT‐2 irrespective of cellular source, and EAAT‐2 is expressed predominantly but not exclusively by astrocytes, our findings are consistent with a major contribution of astrocytic EAAT‐2 to the observed protective effect. Other EAAT‐2‐expressing cell types, including oligodendrocytes, may also play a role in glutamate regulation in the periaxonal space but were not specifically investigated in this study. Notably, in other neurological conditions characterized by primary neuronal loss—such as ALS, ischemia, and epilepsy—or traumatic injury to the CNS, astrocytic EAAT expression has been reported as reduced rather than upregulated (Kim et al. 2011; Li et al. 2014; Alijanpour et al. 2023), a contrast that highlights the specificity of the induction we observe in chronic inflammatory demyelinating lesions.

In EAE, previous reports described mRNA levels of EAAT‐2 in the spinal cord as increased in mice at the onset of the disease compared to control immunized mice (Olechowski et al. 2013). At the peak of the disease, both EAAT‐1 and EAAT‐2 transcription were shown to be increased (Sulkowski et al. 2014) and have been confirmed histopathologically in MS lesions (Werner et al. 2001). Our current data support the upregulation of both EAATs in acute EAE, while we describe for the first time a selective and sustained upregulation for EAAT‐2 in chronic EAE. In MS, lesion morphology is more heterogeneous than in the animal models spanning early active, chronic active, and inactive states (Kuhlmann et al. 2017). Recent spatial and single‐cell analyses of MS lesions have further highlighted marked compartment‐specific astrocyte heterogeneity (Absinta et al. 2021). In particular, inflammatory astrocyte populations have been shown to be enriched at the edges of chronic active lesions, whereas lesion cores display distinct glial states with less inflammatory activation. These findings suggest that astrocytes undergo lesion compartment‐dependent functional reprogramming rather than uniform expansion across lesion types. Within this framework, the increased EAAT expression observed in our study may reflect a functionally distinct astrocyte response rather than simply differences in astrocyte abundance.

However, the current study was not designed to define astrocyte subpopulations and does not include markers such as Lcn2, C3, or other state‐specific transcriptional or protein signatures. Therefore, we cannot determine whether EAAT‐2 upregulating astrocytes identified here correspond to a distinct reactive astrocyte subtype. C3‐expressing astrocytes, which are induced by activated microglia and have been shown to lose typical homeostatic functions including glutamate regulation (Liddelow et al. 2017), are present in MS demyelinating lesions, suggesting that the EAAT‐2‐upregulating astrocytes observed here may represent a phenotypically distinct population. By contrast, Lcn2‐expressing reactive astrocytes have been identified in the active rim of MS lesions in human post‐mortem tissue and shown to exert protective effects in a combined cuprizone/EAE mouse model (Gasterich et al. 2022), though whether they overlap with the EAAT‐2‐upregulating population described here remains to be clarified. Notably, McCallum et al. (McCallum et al. 2026) recently showed that Ccn1‐expressing astrocytes accumulate specifically around white matter lesions in peak and chronic EAE and are elevated in both MS lesions and lesion‐remote NAWM—a pattern that closely mirrors our own observations for EAAT‐2. This is also consistent with the modest gradient in EAAT expression between lesion and NAWM we observed in human tissue (Figure 6), suggesting that NAWM in progressive MS may already exhibit a degree of astrocyte reactivity that is not captured by conventional lesion classifications.

We analyzed here tissue samples from middle‐aged persons with MS who suffered from a severe and often longstanding course of their disease, which contributed to or caused their early death. In these tissue samples, EAAT‐1 and EAAT‐2 expression was increased and varied according to lesion type and cellularity. It would be of great interest to perform a similar analysis in persons with a more benign and less disabling disease course. However, such brain tissue samples are hardly available. A previous report focusing on glia alterations in lesions of a similar patient cohort found both glutamate transporters significantly increased in a transcriptomic analysis of periplaque white matter, in contrast to normal appearing white matter in those patients (Lieury et al. 2014). In IF, upregulation of EAAT‐1 (EAAT‐2 was not investigated by IF) in the periplaque white matter as well as in the lesion was confirmed. However, as EAAT expression was not quantitatively correlated with markers of axonal integrity, demyelination, or inflammatory activity within the same ROIs, the human tissue data demonstrate an association of EAAT expression with lesion type and cellularity but do not establish a causal relationship with tissue protection or injury. Future studies combining EAAT analysis with quantitative assessment of myelin, axonal integrity, inflammation, and astrocyte‐state markers will be required to determine whether distinct EAAT expression patterns are associated with neuroprotective or pathogenic astrocyte responses in MS lesions.

Previous efforts to reduce excitotoxicity in MS targeted glutamatergic signaling directly: riluzole, which inhibits glutamate release, and memantine, an NMDA receptor antagonist, both attenuated clinical EAE (Paul and Bolton 2002; Gilgun‐Sherki et al. 2003). Neither approach, however, translated successfully to clinical trials in people with MS, as broad suppression of glutamatergic transmission carries a substantial risk of adverse effects when physiological signaling is disrupted (Villoslada et al. 2009; Chataway et al. 2020). Our findings suggest a complementary perspective: the sustained upregulation of EAAT‐2 in chronic EAE lesions, together with altered EAAT expression in human chronic MS tissue, is consistent with an endogenous response that may contribute to limiting excess extracellular glutamate at inflammatory lesion sites without globally impairing glutamatergic function.

Future studies might focus on identifying the sources of excess glutamate. The hypothesis that this is partly due to release from inflammatory cells activated by cytokines is supported by our intravital recordings in acute and chronic EAE, as well as by prior work (Siffrin, Radbruch, et al. 2010; Birkner et al. 2020). Additionally, our findings may prompt investigations into the variability of EAAT expression in pwMS, which could provide insights into the diverse disease courses observed across individuals. Investigating the persistence of EAAT expression in MS lesions, and the factors that promote beneficial astrogliosis, may be particularly relevant in this context. PET (positron emission tomography) radiotracers targeting EAAT‐2 could offer a valuable tool to explore the role of glutamate transporters in human neurodegenerative pathology, including MS (Fontana et al. 2023), and such studies would be interesting to correlate with the survival of neuro‐axonal structures in existing lesions—potentially helping to identify compounds that successfully address the neurodegenerative aspects of MS.

## 4 Material and Methods

### 4.1 Mice

Approval for all animal procedures was granted by the Landesamt für Gesundheit und Soziales Berlin (license G0056/15 for TPLSM experiments and G0009/16 for histological analysis). The breeding of all animals was conducted in‐house under specific pathogen‐free (SPF) conditions at the Max Delbrück Center for Molecular Medicine in Berlin. Both female and male mice were used, with a minimum age of 8 weeks upon entry into the experiments. All procedures adhered to the European Union guidelines for the care and use of experimental animals.

Mouse lines iGluSnFR [B6;129S‐Igs7tm85.1(tetO‐gltI/GFP*)Hze/J] (Madisen et al. 2015), and R26‐LNL‐tTA [B6.129P2(Cg)‐Gt(ROSA)26Sortm1(tTA)Roos/J] (Wang et al. 2008) were purchased from the Jackson Laboratory (USA) and crossbred with the reporter strain Nex‐Cre (Goebbels et al. 2006), kindly provided by Klaus‐Armin Nave (MPI Göttingen, Germany). Mouse line B6.Thy1.CertnL15 (Heim et al. 2007) was generously provided by Oliver Griesbeck (MPI Munich), B6.tdRFP^fl/fl^ and B6.acRFP (Luche et al. 2007) were given by Hans‐Jörg Fehling (University Ulm, Germany).

For bone marrow chimeric mice, recipients were lethally irradiated with a split dose of 1100 cGy. Donor animals were euthanized by neck dissection, and bone marrow cells were harvested by flushing the femur and tibia, followed by dissolution and MACS depletion of CD90+ T cells using CD90 beads (Miltenyi Biotec, Germany). Approximately 8 h post‐irradiation, recipients were reconstituted with sex‐matched 5–10 × 10^6^ T cell‐depleted donor bone marrow cells. Mice were given 0.01% Enrofloxacin (Baytril, Bayer HealthCare) in their drinking water for 4 weeks, and engraftment occurred over at least 8 weeks before EAE induction.

### 4.2 Experimental Autoimmune Encephalomyelitis

Mice that were at least 16 weeks old (8 weeks after engraftment) were immunized with a subcutaneous injection of 125 μg myelin oligodendrocyte glycoprotein (MOG)35–55 peptide (obtained from Rudolf Volkmer‐Engert, Charité Universitätsmedizin Berlin). This was emulsified in 400 μg *Mycobacterium tuberculosis* H‐37Ra and complete Freund's adjuvant (Sigma‐Aldrich). Two and 24 h later, the mice were given an additional intraperitoneal injection of 300 ng pertussis toxin dissolved in phosphate‐buffered saline (PBS, Gibco).

The mice were weighed daily, and their clinical condition was assessed using a scoring system that evaluated four separate symptoms, each with varying degrees of severity. Points were assigned as follows: tail paresis (0.5 points) or tail plegia (1.0 point), weakness of righting reflex (0.5 points), hind limb paresis (1.0 point) or hind limb plegia (2.0 points), and forelimb paresis (0.5 points) or forelimb plegia (1.0 point). The total score was determined by adding the individual points together.

Mice that reached a score of 2 or higher after the onset of clinical signs were given corticosteroid treatment via intraperitoneal injection of 1.6 mg Methylprednisolone in 200 μL 0.9% NaCl for 3–5 days to induce remission of experimental autoimmune encephalomyelitis (EAE) (Schmidt et al. 2000; Chan et al. 2008). Mice that reached scores of 3.5 or higher or lost more than 20% of their initial weight were euthanized in accordance with animal welfare protocols.

### 4.3 Immunofluorescence of Mouse Spinal Cord Tissue

Mice were euthanized with an intraperitoneal injection of pentobarbital (RELEASE, 800 μg/g) or ketamine (Ketanest, 320 μg/g) and xylazine (Rompun, 5 μg/g). The mice were then perfused transcardially with 50 mL of phosphate‐buffered saline (PBS) followed by 35 mL of 4% solution of ultrapure formaldehyde (16% FA; Polysciences) in PBS for fixation. Spinal cord samples were collected and incubated overnight in 2% FA at 4°C. The next day, the FA was removed and replaced with PBS. Samples were stored at 4°C until dehydration, which was conducted in sucrose solutions of increasing concentrations (10%, 20%, and 30%) for 24 h each.

The tissue samples were embedded in Tissue‐Tek O.C.T. Compound (Sakura) and slowly frozen using dry ice, then stored at −80°C until being cut into 8 μm‐thick cross sections with a cryostat microtome. The sections were mounted on glass slides and stored at −80°C until use. For staining, the sections were thawed for 30 min at room temperature and rehydrated in PBS for 20 min. Antigen retrieval was achieved by heating the slices in citrate buffer (pH 6.0) for 2 min in a pressure cooker. After cooling to room temperature, a blocking solution of 100 μL PBS, 1% bovine serum albumin (BSA), and 10% goat serum was applied for 20 min to reduce nonspecific binding.

Slices were washed three times with PBS containing 1% BSA for 5 min each. The tissue was permeabilized for 10 min in 100 μL PBS with 0.4% Triton‐X‐100 at room temperature. After a second rinsing cycle, primary antibodies were applied in a solution of PBS, 1% BSA, and 2% goat serum, then incubated overnight at 4°C in the dark. The following day, another rinsing cycle was conducted before incubating the secondary antibodies for 1 h at room temperature in the dark.

After washing the samples as previously described, the sections were embedded in DAKO medium and covered with a cover glass. The sections were allowed to dry for 5 min and were then stored at 4°C in the dark. Control IF samples were prepared using the same staining procedure but without primary antibodies (Table 2).

**TABLE 2:** Antibodies for immunofluorescence staining of mouse spinal cord tissue and human tissue.

Antibody	Fluorophore	Clone	Company	Cat. No.	Species	Reactivity	Dilution
GFAP*	Alexa Fluor 488	GA5	Thermo Fisher Scientific	53‐9892‐82	Mouse	Hu, ms, rat	1:200
GFAP**	Brilliant Violet 421	2E1.E9	Biolegend	644710	Mouse	Hu, ms, rat	1:100
GFAP***	PE	REA335|1B4	Miltenyi Biotec	130‐118‐351	Human cell line	Hu, ms, rat	1:50
HLA‐DR, DP, DQ	PE	REA332|Tu39	Miltenyi Biotec	130‐120‐715	Human cell line	Hu	1:50
EAAT‐1	Unconjugated	Polyclonal	Elabsience	E‐AB‐31258	Rabbit	Hu, (ms)	1:100
EAAT‐2	Unconjugated	Polyclonal	Abcam	ab41621	Rabbit	Hu, ms, rat	1:100
NF‐L	Alexa Fluor 647	NFL3	Biolegend	845,907	Mouse	Hu, ms, rat	1:100
MBP	PE	REA1154	Miltenyi Biotec	130‐120‐342	Human cell line	Hu, ms, rat	1:50
CD45	PE	30F11	Miltenyi Biotec	130‐117‐498	Rat	ms	1:20
Anti‐rabbit	Alexa Fluor 594	Polyclonal	Thermo Fisher Scientific	—	Goat	Rabbit	1:400

*Note:* * used for human tissue, ** used for mouse tissue, *** used for MACSIMA experiments.

### 4.4 IF Staining of Human Brain Tissue

Human post‐mortem brain tissue was obtained from the Netherlands Brain Bank (NBB; RRID: SCR_013841), Netherlands Institute for Neuroscience, Amsterdam (open access: http://www.brainbank.nl/). All material had been collected from donors for or from whom written informed consent for brain autopsy and the use of material and clinical information for research purposes had been obtained by the NBB (project no. 1034; internal ethical approval EA1/408/16). Human ALS tissue samples were provided by the Biobank at the Department of Neuropathology at Charité (local ethical approval EA1/144/13). The cryo‐preserved tissue pieces were snapfrozen and stored at −80°C. Using a cryostat, the samples were sectioned into 8 μm‐thick slices. Lesions were staged according to https://link.springer.com/article/10.1007/s00401‐018‐1818‐y by a neuropathologist at NBB or Charité on a mirror FFPE block. For immunofluorescence staining, the sections were thawed at room temperature for 30 min and then fixed with 4% formaldehyde for 10 min. Next, the samples were washed and incubated in PBS for 20 min at room temperature.

Blocking solution (100 μL PBS, 1% bovine serum albumin (BSA), and 10% goat serum) was applied for 20 min before the addition of primary unconjugated antibodies (Table 2) in a solution of PBS, 1% BSA, and 2% goat serum. Permeabilization was carried out for 10 min with 100 μL PBS plus 0.4% Triton‐X‐100 at room temperature, then the samples were incubated with the primary antibodies overnight at 4°C. The next day, samples were washed three times for 5 min each with PBS containing 1% BSA. Secondary antibodies were then incubated for 1 h at room temperature in the dark in a solution of PBS, 1% BSA, and 2% goat serum.

Finally, DAPI (1:4000) in PBS and 1% BSA was applied for 10 min at room temperature before embedding the slides in DAKO medium and covering them with a cover glass.

### 4.5 Wide‐Field Microscopy and Image Analysis

Two‐dimensional fluorescence intensity images were captured using a Leica wide‐field microscope or an Axioscan 7 (Zeiss) slide scanner. The filter sets chosen to image DAPI and the secondary antibodies for fluorescently labeling tissue sections are listed in Table 3. A 40× dry objective was used to image areas of 320.66 × 239.57 μm (1392 × 1040 pixels) on mouse spinal cord sections. For human cryosections, a 20× dry objective was used. Additionally, large‐area views of each sample were obtained by stitching together around 100 tile images.

**TABLE 3:** Excitation and detection filters for wide field imaging.

DAPI and secondary antibodies	DAPI	Alexa Fluor 488	Alexa Fluor 594 PE	Alexa Fluor 647
Excitation filter (nm)	320–400	440–520	535–557	560–680
Dichroic beamsplitter (nm)	400	505	565	660
Emission filter (nm)	430–510	497–557	560–640	625–775

The images, consisting of three or four channels (depending on the antibody combinations) and one *z*‐plane, were saved in LIF file format and then converted to TIFF format using ImageJ/Fiji software for quantitative analysis. Individual areas used for quantitative analysis were further cropped to a size of 158 × 158 μm (700 × 700 pixels).

To quantify the fluorescence intensities, we used ImageJ/Fiji (https://imagej.net/software/fiji) and Volocity software (Quorum Technologies Inc., Canada, https://www.volocity4d.com/). Regions of interest (ROIs) in EAE tissue were defined based on inflammatory infiltration (CD45), increased cellularity (DAPI), and reactive gliosis (GFAP). MBP staining provided complementary information on myelin integrity but was not used as the primary criterion for lesion delineation. The ROIs measured 50 × 50 μm and were symmetrically defined in the modified regions and contralateral anatomical structures, respectively outside the lesion in human tissue.

The cropped images were imported into Volocity, where fluorescent objects were segmented using a minimum size of 10 μm^2^ in the GFAP channel (our pan‐astrocyte marker) and 2.5 μm^2^ in the NF channel (axonal marker). The mean fluorescence intensity in the EAAT‐1 and EAAT‐2 channels was then evaluated within the segmented areas. These values were compared between lesion areas and contralateral control areas.

### 4.6 Statistical Analysis of IF

Statistical analysis was performed using GraphPad Prism 10 or 11 (GraphStats Technologies, USA). Mean fluorescence intensity (MFI) values were measured within GFAP‐segmented regions in lesion areas (LA) and corresponding contralateral normal‐appearing white matter control areas (CA) within the same tissue section (one to four area pairs per section, depending on the number of lesion sites identified). Outliers were identified and removed using the ROUT method (*Q* = 1%). MFI values were then normalized to the median of the corresponding CA (normalized value = MFI/median CA). The median of normalized values was calculated per ROI, and the mean of these regional medians was used as a single value per animal. Statistical comparisons between groups (naïve, acute EAE, chronic EAE) were performed using the Brown‐Forsythe and Welch ANOVA with Dunnett's T3 multiple comparisons test. Test selection was based on the normality of the data (Shapiro–Wilk test, *p* > 0.05 for all groups) combined with evidence of unequal variances (Brown‐Forsythe and Bartlett's tests), for which Welch ANOVA is the appropriate parametric test. A *p* value < 0.05 was deemed statistically significant.

For NF‐L evaluation, the NF‐L‐positive area per ROI was calculated as the sum of all NF‐L object areas (μm^2^) identified within each ROI divided by the total ROI area (24,964 μm^2^; 158 × 158 μm), yielding a dimensionless area fraction as a measure of axonal density. Outliers among individual NF‐L object areas were identified and removed using the ROUT method (*Q* = 1%) prior to summation. Values were normalized to the corresponding control area (NAWM) by dividing the mean of lesion area values by the mean of NAWM values per animal, yielding one ratio per mouse. Group comparisons were performed using the Brown‐Forsythe and Welch ANOVA with Dunnett's T3 multiple comparisons test, consistent with the approach used for EAAT quantification.

### 4.7 MACSima Imaging

Human NBB tissue was analyzed using the MACSima Imaging System (Miltenyi Biotec). Cryosections (8 μm), stored at −80°C, were thawed for 30 min at room temperature and rehydrated in PBS for 20 min. Sections were subsequently fixed with freshly diluted, methanol‐free 4% paraformaldehyde (PFA) for 10 min at room temperature, followed by washing in PBS for 10 min. Subsequent sample processing, cyclic immunofluorescence staining, image acquisition, and processing were performed by Miltenyi Biotec according to the manufacturer's standard MACSima Imaging Cyclic Staining (MICS) protocol. The following antibodies were used: anti‐HLA‐DR‐DP‐DQ (clone REA332, PE‐VIO‐670; Miltenyi Biotec), anti‐MBP (clone REA1154, PE; Miltenyi Biotec), and anti‐GFAP (clone REA335, VIO‐R667; Miltenyi Biotec).

### 4.8 Operation Procedures for Intravital Microscopy

The animal experiments were conducted in compliance with the regulations and guidelines set by the local authorities (G0056/15 and G009/16), Operational procedures were conducted following the methods described in a previous study (Siffrin et al. 2009) with adaptations. In short, animals were anesthetized via intraperitoneal (i.p.) injection of a combination of midazolam (5 μg/g body weight), fentanyl (0.05 μg/g), medetomidine (0.5 μg/g). Mice were then intubated through the oral cavity with the plastic part of an IV catheter (Vasofix 20G, Braun, Germany) and ventilated using a Minivent (Type 845, Hugo Sachs Elektronik‐Harvard Apparatus) with a volume of 200–250 μL for females and up to 300 μL for males. The depth of anesthesia was monitored by continuous measurement of exhaled CO_2_ levels using a CI‐240 Microcapnograph (Columbus Instruments, USA). Anesthesia was then switched to a mixture of oxygen and nitrous oxide (1/3 O_2_, 2/3 N_2_O) along with Isoflurane (1%–2.5%). After establishment of ventilation anesthesia, midazolam and medetomidine were antagonized with flumazenile (0.5 μg/g) and atipamezole (2.5 μg/g) respectively. The animal was fixed in a stereotactic frame on a heated platform during the whole imaging process. The body temperature of the animals was measured and maintained within the range of 34°C–37°C. ECG was continuously monitored. The skin and muscles covering the occipital part of the skull and upper cervical spinal cord were incised and carefully dislocated to expose the lower brain stem between skull and first cervical vertebra. The dura was incised and removed. The exposed spinal cord was covered with a cover glass which was fixed with thin layer of pre‐warmed 1% Agarose (in isotonic electrolyte solution, Sterofundin) solution. Anesthesia depth was controlled by monitoring the exhaled CO_2_ and the ECG.

### 4.9 Two‐Photon Laser Scanning Microscopy

TPLSM was conducted on the brainstem of control and EAE‐induced transgenic mice expressing a genetically encoded Ca^2+^ indicator (GECI) in neurons and their processes, as well as in mice expressing the glutamate biosensor iGluSnFR. The biosensor functions based on a conformational change of a glutamate‐binding protein (the *E. coli* ABC transporter for glutamate and aspartate) which induces in its turn a change in the chromophore environment of a circularly permuted GFP (Marvin et al. 2013). NEX‐Cre‐mediated recombination has been shown to selectively target postmitotic neurons, with no detectable recombination in astrocytes, oligodendrocytes, or GABAergic interneurons in the extensively characterized telencephalon (Goebbels et al. 2006).

Ca^2+^ activation was detected via fluorescence lifetime imaging (FLIM) by measuring the reduced lifetime of the donor (Cerulean) in the Cerulean/YPet FRET thy1‐CerTn‐L15 biosensor. Activation of the glutamate biosensor was monitored by measuring the increased fluorescence intensity (FI) in the GFP channel. The imaging setup consisted of an upright Trimscope II multiphoton microscope (LaVision Biotec GmbH, Germany) that uses a pulsed Ti:Sapph laser (Chameleon, Coherent, USA) tuned at 860 nm. The laser emission was split using a polarizing beamsplitter, with one beam going directly to the objective for the Cerulean or iGluSnFr excitation, and the other beam pumping an optical parametric oscillator to generate the 1110 nm wavelength used for the excitation of tdRFP expressed in hematopoietic cells. Imaging was done using a 20×/NA 0.95 water dipping Olympus objective or a 25×/NA1.1 Nikon water dipping objective. Detection was collected at 475/50 nm for Cerulean or to 520/40 nm for iGluSnFr and (if necessary) it was split with a long pass 560 nm dichroic to be collected at 628/40 nm for tdRFP. Fluorescence lifetime measurements of Cerulean were done using the time correlated single photon counting (TCSPC) method and a hybrid detector. The iGluSnFR fluorescence intensity was detected with the same hybrid detector, while the detection of the red fluorescent protein intensity was done using a sensitive GaAsP detector, simultaneously with the acquisition in the Cerulean or GFP channel. Time lapse acquisition of brainstem volumes (i.e., *z* stacks; usually between 19 and 30 *z* steps per time point) was conducted for at least 2 h (usually 2 min interval between *z* stacks), starting with a baseline acquisition followed by the addition of the different drugs to the fluid topping the imaging area. The fluid was kept fresh by slow exchange with a peristaltic pump of pre‐warmed isotonic electrolyte solution (Sterofundin). For in vivo imaging, inflammatory lesion areas were identified by the local accumulation of tdRFP‐labeled peripheral immune cells within the brainstem, which served as a real‐time marker of inflammatory infiltration.

### 4.10 TPLSM Data Analysis: FLIM Analysis (Cerulean in the thy1‐CerTn‐L15 FRET Sensor)

We implemented the analysis of the multidimensional ome.tiff FLIM images acquired using the Imspector software (LaVision Biotec GmbH, Germany) in the open‐source software FLIMfit (version 5.0.3, https://flimfit.org/) (Warren et al. 2013). Fluorescence intensity decay curves were fit with a double exponential model using IRF (instrument response function) deconvolution. The IRF was measured using the second harmonic generation signal of urea crystals deposited on a microscope slide. The weighted mean lifetime reported in the paper was calculated as:

$$\tau_{\text{mean}}=\frac{\alpha_{\text{long}}\tau_{\text{long}}^{2}+\alpha_{\text{short}}\tau_{\text{short}}^{2}}{\alpha_{\text{long}}\tau_{\text{long}}+\alpha_{\text{short}}\tau_{\text{short}}}=f_{\text{long}}\tau_{\text{long}}+f_{\text{short}}\tau_{\text{short}}$$

where *α* is the amplitude and *τ* is the decay time for the long and short components in the fit, and *f*_long_ and *f*_short_ are the fractions of the two lifetime components (with *f*_long_ + *f*_short_ = 1). Mean lifetime (raw) images in a 32‐bit format were exported from FLIMfit and used for further analysis in ImageJ/Fiji.

To remove artifacts in the Cerulean channel due to spectral cross‐talk and bleed‐through of the red fluorophore, masks were generated using thresholded intensity images from the tdRFP channel (628/40 nm) and the binarization algorithm in ImageJ/Fiji. The FLIM image sequences (consisting of z planes and time points) were multiplied with their respective mask sequences and used for further analysis. Movement correction was performed using the “3D drift correct” plugin (Parslow et al. 2014).

To exclude artifacts from fitting pixels with low counts and tissue autofluorescence, lifetime images were analyzed using thresholds between 1.4 and 3.2 ns. Sets of 8–12 *z* planes with similar anatomical structures were selected at each time point and analyzed along the time axis. The median lifetime values of each z‐plane were used to calculate a single mean value and standard deviation (SD) for each time point.

For graphical presentation, outliers were removed in GraphPad Prism. The relative variation of the mean lifetime value over time was calculated by subtracting the mean FLIM value of the final 20 min before starting treatment.

### 4.11 TPLSM Data Analysis: Intensity Analysis (GFP in the iGluSnFr Biosensor)

GFP fluorescence was detected using a 520/40 nm band filter in front of the FLIM hybrid detector. Data were loaded into the FLIMfit software, and intensity images containing the total fluorescence counts per pixel were exported as 8‐bit TIFF files for further analysis in ImageJ/Fiji. The intensity image series were multiplied with a mask derived from the tdRFP channel (628/40 nm) similarly to the Cerulean analysis. Images were corrected for movement using the “3D drift correct” plugin (Parslow et al. 2014). A control region (mostly a vessel) was used as region of interest (ROI) to identify a mean background Fluorescence F in each image. This mean F was used to calculate with ImageJ the Δ*F*/*F*, where Δ*F* = pixel intensity—mean background intensity and *F* = mean background intensity. These image planes were analyzed for mean Δ*F*/*F* per image frame and recording. The single values represent the mean of the mean Δ*F*/*F* of each *z*‐plane (pooled out of individual mice). For some mice different imaging was recorded and analyzed.

## Author Contributions

L.R., A.M., and V.S. designed the research project and the experiments and wrote the manuscript. J.C. supported experiments and data analysis. I.M. contributed to the development of the FLIMit software and provided critical advice. M.A., K.R., and H.R. supported experiments and performed analyses.

## Funding

Grants from the DFG (German Research Foundation) to VS (Si 1886/1‐1, Si 1886/3‐1, and Si 1886/4‐1) and the Gemeinnützige Hertie‐Stiftung to VS (Hertie MyLab programme) and Luise Reukauf (MedMS, P1190041) supported the project.

## Conflicts of Interest

The authors declare no conflicts of interest.

## Supporting information


**Figure S1:** Identification of inflammatory lesion areas in murine EAE spinal cord.

**Figure S2:** Genetically encoded biosensors and fluorescent expressed in mice for intravital imaging.

**Figure S3:** Axonal Ca^2+^ dynamics in chronic EAE and following EAAT blockade in naive mice.

**Figure S4:** MBP and HLA‐DR characterization of human post‐mortem MS white matter lesions.

**Figure S5:** Regional analysis of EAAT‐1 and EAAT‐2 expression in a representative chronic MS lesion and comparison with ALS white matter.

## Data Availability

The data that support the findings of this study are available on request from the corresponding author. The data are not publicly available due to privacy or ethical restrictions.
